# Utilization of refuse-derived fuel in industrial applications: Insights from Uttar Pradesh, India

**DOI:** 10.1016/j.heliyon.2024.e41336

**Published:** 2024-12-18

**Authors:** Utsav Sharma, Dayanand Sharma, Amit Kumar, Tushar Bansal, Ankit Agarwal, Shudhanshu Kumar, Abid Hussian, Hesam Kamyab, Moinul Haq

**Affiliations:** a Department of Civil Engineering, Sharda School of Engineering and Technology, Sharda University, Greater Noida, 201310, India; b Clean India Environment Pvt Ltd, Gurgaon, Haryana 122018, India; c Department of Mechanical Engineering, Maulana Azad National Institute of Technology, Bhopal 462003, India; d Department of Civil and Environmental Engineering, Carleton University, Ottawa, Canada; e UTE University, Faculty of Architecture and Urbanism, Architecture Department, TCEMC Investigation Group, Calle Rumipamba S/N and Bourgeois, Quito, Ecuador; f Department of Biomaterials, Saveetha Dental College and Hospital, Saveetha Institute of Medical and Technical Sciences, Chennai, 600077, India; g The KU-KIST Graduate School of Energy and Environment, Korea University, 145 Anam-Ro, Seongbuk-Gu, Seoul, 02841, Republic of Korea; h Interdisciplinary Research Center for Construction and Building Materials, Research Institute, King Fahd University of Petroleum & Minerals, Dhahran, 31261, Saudi Arabia

**Keywords:** Refuse-derived fuel, Municipal solid waste, Waste management, Sustainable energy, Environmental impact, Industrial application

## Abstract

Urbanization and population growth in India have quickened, leading to an annual generation of around 62 million tonnes of municipal solid waste (MSW). Improper management of organic waste presents a major environmental problem due to air and water pollution, soil contamination and greenhouse gas production. This research aims to develop refuse-derived fuel (RDF) as a viable option, converting waste into a high-calorific energy carrier for industrial use. The RDF samples were collected from five strategic locations in Uttar Pradesh: Morta Site, Pipeline Site, and Sector 146 Noida, covering various waste compositions found at these landfill sites. Proximate and ultimate analyses of the RDF prepared from these sources were conducted, followed by in-depth Thermogravimetric Analysis (TGA) to validate its suitability as a potential feedstock. Careful waste segregation and treatment for better fuel quality can help minimize the difference in calorific values between different sites. Based on RDF tests, the waste-to-energy technology can divert over 30 % of solid waste from landfills and cut greenhouse gas emissions by as much as 25 % compared to traditional disposal methods. Unlike RDF, which is part of the replacement line for coal in industrial furnaces such as thermal power plants, it eliminates over 15 % and 20 % of sulfur dioxide (SO_2_) and nitrogen oxides (NOx). Ensuring that RDFs support sustainable energy technologies and align with circular economy principles, the study's results could enhance energy efficiency in waste management and complement environmental policy goals across all states in India and worldwide.

## 1 Introduction

India, recognized as one of the world's most rapidly advancing economies and now the most populous nation, is grappling with serious environmental issues stemming from swift urban development and population growth. The National Commission on Population Ministry of Health & Family Welfare (2020) forecasts that by 2036, India's population will soar to roughly 1.52 billion [1]. According to the latest census, India's urban populace has been growing significantly, with figures rising to 377 million in the 2011 Census and anticipated to reach 600 million by 2031 [1,2]. Economic growth and urbanization generally increase solid waste generation [2]. This has compounded environmental issues, mainly due to the need for more waste management infrastructure. A report by The Central Pollution Control Board (CPCB) records that India generates 62 million tonnes of Municipal Solid Waste (MSW) annually; only around 75–80 % is collected and approximately between 22 and 28 % is processed [3]. Municipal solid waste (MSW) is a major environmental threat for non-scientific disposal as it affects air and water pollution, soil contamination, and global warming [3,4]. The old way of waste management, the traditional methods of landfilling and open dumping, are no longer sustainable mainly because they deplete resources that have become scarce, and their ecological repercussions cause harm to all. Methane gas, 25 times more harmful to the environment than carbon dioxide by volume, is emitted from landfills when organic waste decomposes. Moreover, landfills being the final disposal destinations may lead to dangerous health threats since contaminated leachate generated due to garbage dumping infiltrates through soil and pollutes groundwater, posing life-threatening risks in neighbouring vicinities [2,4].

There is a need for such an alternative that can be converted to Refuse-Derived Fuel (RDF) in thermochemical treatment processes and, thus, the best possible way method adaptable for managing municipal solid waste (MSW). RDF is produced through the systematic separation, confinement and treatment of waste in landfill sites to get the highest quality fuel and performance. These processes condition the material for energy use in industrial boilers, cement kilns or power plants. Providing RDF is beneficial in many ways for the environment and economic purposes [6]. It cuts down landfill use, hence lowering methane emission and soil contamination. In addition, replacing some of the coal used in industry processes like cement production for RDF can reduce emission rates of pollutants such as SO₂ and NOₓ or others [7].

RDF is also necessary to save waste as a resource and support a circular economy by reducing reliance on non-renewable locally-based energy systems [8]. which support all existing co-processing of municipal solid waste solutions. Hemidat et al. [9] Assessed the RDF had been used by the cement industries in the Jordan. They found that RDF could justify around 21.2 % of plant process heat load, resulting in production cost reductions and improved energy performance. Additionally, Kumar et al. [10] and Zhang et al. [11] has been shown at the industrial level that integrating RDF into processes with increasing temperatures (cement kiln) would reduce CO₂ emissions by up to 25–30 % versus traditional fossil fuels [12].

RDF is additionally established to be a potential energy recovery fuel; calorific values of RDF vary from 15 to 25 MJ/kg depending on source and waste composition [12]. RDF also results in less CO₂ emissions and fuel costs. Tahiru et al. [13] conducted an Life Cycle Assessment (LCA) to analyze RDFs from Municipal Solid Waste (MSW) in Accra and Kumasi/Ghana. According to the results, plastic-wood fuels with a heating value of 28.66–30.24 MJ/kg exhibited over $10 % savings in coal used for industrial applications. The study by Santos et al. [14] examined the characteristics of different material streams, MSW, RDF and stove ash/bottom ash, and the life cycle assessment of a waste-to-energy plant. Their study identified 60–65 % RDF from processing of 1 million tons of MSW as an optimum co-processor that can be combusted to generate electricity with capacity at around the level of 0.45 MW, respectively.

LCA studies indicate that waste-to-energy plants maximize energy recovery while ensuring the safe disposal of inert materials in landfills, achieving a relatively low Global Warming Potential (GWP) of 332 kg CO₂ eq. However, most research on RDF has been studied in developed regions, particularly in Europe and North America, where advanced waste management infrastructure is well established. In contrast, regions like Uttar Pradesh (U.P.), which face substantial waste management challenges, have yet to be studied in sufficient detail. U.P., India's most populous state, generates over 15,000 tonnes of municipal solid waste daily, yet only a fraction of this waste is processed, with the majority being sent to landfills [3,4]. The state's rapid urbanization, the population density of 828 people per square kilometre (as per the 2021 census), and burgeoning industrial base make it an ideal case study for exploring the potential of RDF [4,5]. While previous studies have focused on the technical feasibility of RDF in regions with advanced waste management systems, there is a critical lack of research that addresses the challenges and opportunities for RDF in areas like the U.P., where waste segregation and processing infrastructure are still developing. This study addresses a significant gap in the existing body of knowledge by examining RDF production and characteristics across five distinct locations in Uttar Pradesh.

This study discusses RDF in detail via empirical data on production processes, utilization forms and environmental impacts. This paper aims to outline the advantages and disadvantages of RDF as a fuel source, utilizing comprehensive data from the Central Pollution Control Board (CPCB) and other relevant sources. The case study presents accurate RDF data from five locations in Uttar Pradesh, India, within the framework of India's National Action Plan on Climate Change (NAPCC). This plan emphasizes the promotion of sustainable technologies to reduce carbon emissions and enhance energy security. The U.P. could meet much of its climate goals by cutting waste headed to landfills and swapping out coal (or other fossil fuels) for RDF in power plants instead. This research further adds to the global conversation for sustainable waste management. It is in line with the United Nations Sustainable Development Goals (SDG), predominantly SDG 12 (Responsible Consumption & Production) and SDG 13 (Climate Action). RDF is a cost-effective solution to reduce rubbish sent to landfill by up to 60 % more than traditional waste handling arrangements, reducing environmental consequences of disposal and improving energy recovery capacity and industrial efficiency.

## 2 Generation, composition, and management of MSW

Knowing waste management techniques and MSW generation helps in understanding RDF production. MSW is a complex material that contains heterogenous mixtures of different substances. The generation and composition of MSW are essential for designing competent waste management actions. Waste generation and composition differ significantly among countries based on economic development, urbanization, consumer incomes, etc. The global solid waste generation was about 2.01 billion tons per annum, with an average of 0.74 kg per day per capita in urban areas [2,4,5]. According to CPCB data, India produced 160,038 tonnes of waste daily in 2022 [3].

**Fig. 1:**
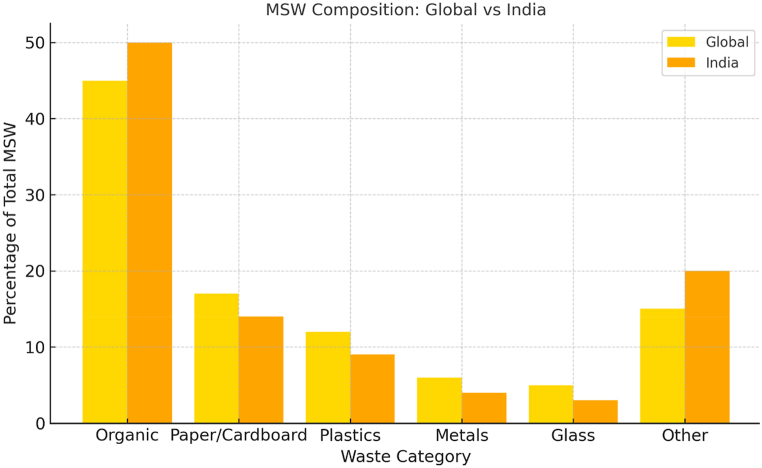
MSW composition global and India [3,4].

The MSW fraction globally and in India is shown in Fig. 1. From Fig. 1, it is observed that organic waste comprises 44 % of global MSW, followed by paper and cardboard (17 %), plastics (12 %), metals (4 %), glass (5 %), and other materials at 18 %, based on data (Kaza et al., 2018). Similarly, organic waste in India is the same proportion of MSW (50 %, ranging from 40 to 60 %) as in Bangladesh. This value indicates that the share of organic waste in India is higher than that of other types of waste, which is likely due to more food waste and agricultural residues. Due to consumption patterns, the prevalence of plastics (in terms of weight percentage) is lower than that of metals in India. Plastics constitute around 10 % w/w of MSW, with metals making up the majority of the composition globally, highlighting differences in recycling practices on an international scale. From the observations mentioned above, it is found that the composition of MSW is a critical factor influencing waste management strategies, including the feasibility of RDF production. In context to this study, current waste management practices in Uttar Pradesh remain fragmented and vary significantly between cities. Waste collection systems need to be improved, with coverage rates ranging from 70 % in larger cities to less than 50 % in smaller municipalities. The predominant disposal method in the state is landfilling, with open dumping still being practised in many areas due to a lack of infrastructure for more advanced treatment technologies like incineration or anaerobic digestion.

Regarding waste treatment, only a tiny fraction of the MSW generated is subjected to formal processing, with composting facilities operating at limited capacity and recycling rates remaining below 20 %. RDF production has been identified as a potential solution to waste disposal and energy generation challenges in cities like Kanpur and Ghaziabad, where industrial demand for alternative fuels is higher [15]. However, the effectiveness of RDF production is heavily influenced by the composition and quality of the waste collected and the technologies employed in waste processing. For instance, RDF derived from fresh waste through MBT processes typically has higher calorific values (up to 22 MJ/kg) than RDF produced from landfill mining, which tends to have lower energy content due to the degradation of organic materials [15,16]. The state's dependence on traditional waste management practices, such as landfilling and low recycling rates, highlights a significant inefficiency. This issue could be addressed by adopting integrated waste management systems emphasising RDF production, composting, and recycling.

RDF presents numerous advantages; however, waste management in Uttar Pradesh is hindered by inadequate infrastructure and a large volume of unsegregated waste, with many residents unaware of source segregation. These challenges exacerbate improper municipal solid waste management. Nonetheless, rising industrial demand offers a chance to enhance RDF production and customize waste treatment solutions based on regional characteristics.

### 2.1 Waste management techniques

**Fig. 2:**
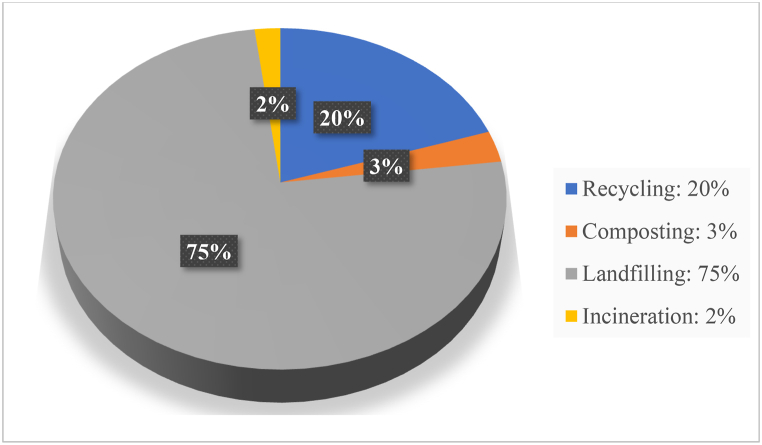
Indian waste management scenario (CPCB [3]).

**Fig. 3:**
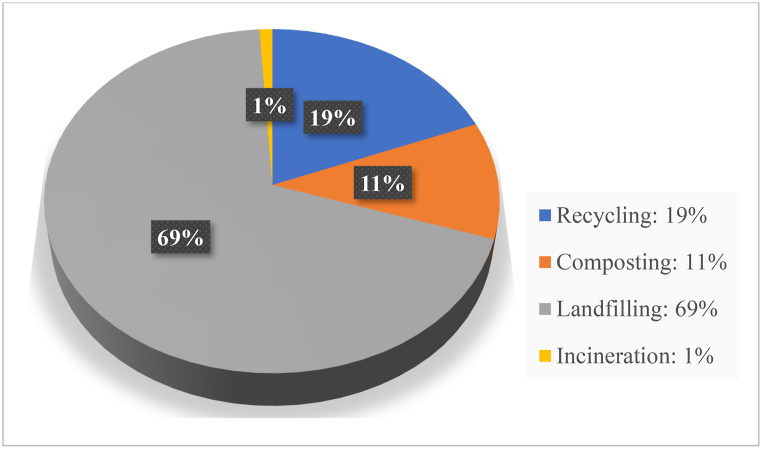
Global waste management scenario (Kaza et al. [8].

To address health risks linked to landfill waste, effective waste management strategies are essential. Reuse involves repurposing waste into new products, conserving resources and reducing landfill reliance (refer Fig. 2, Fig. 3) [16]. Composting is a natural decomposition process that transforms organic waste into valuable soil amendments, restoring nutrients to the soil [17]. Incineration turns waste into useful energy while employing pollutant controls to minimize air pollution [18]. Landfilling involves burying waste in designated areas to mitigate pollution risks [19]. Advanced methods like anaerobic digestion convert organic waste into biogas and digestate [19], while pyrolysis and gasification thermally transform waste into synthetic fuels [20]. Table 1 summarises the advantages and limitations of these waste management methods. Fig. 2, Fig. 3 present pie charts illustrating the global and Indian distribution of waste management practices, indicating that landfilling remains the most widely used method.

**Table 1:** Merits and demerits of waste management techniques.

Technique	Merits	Demerits	References
Recycling	Reduces landfill waste, conserves natural resources, and lowers energy consumption compared to producing new materials.	Requires sorting and processing infrastructure, potential contamination issues, and not all materials are recyclable.	[16]
Composting	Produces nutrient-rich soil amendments, reduces landfill use, and minimizes greenhouse gas emissions from organic waste decomposition.	Needs space and time for the process, can produce odors, and may attract pests if not managed properly.	[17]
Incineration	Reduces waste volume significantly, generates energy, and can handle various waste types.	Emits pollutants if not properly controlled, high operational costs, and ash disposal issues.	[19]
Landfilling	Handles large amounts of waste, relatively low initial cost, and can be designed to protect the environment with liners and leachate management.	Long-term environmental impact, potential for groundwater contamination, and methane emissions contributing to climate change.	[20]
Anaerobic Digestion	Produces biogas for energy, reduces landfill waste, and generates nutrient-rich digestate as a byproduct.	Requires controlled conditions, initial setup costs can be high, and not all waste is suitable for digestion.	[21]
Pyrolysis/Gasification	Converts waste into valuable products like synthetic fuels, reduces landfill volume, and can handle various waste types.	High technological and operational costs, complex processes, and potential emissions if not managed properly.	[11]
Waste-to-Energy	Reduces waste volume, generates renewable energy, and can decrease reliance on landfills.	High initial costs, potential emissions, and requires continuous waste supply to be cost-effective.	[12,14]

However, much global waste, including in India, still needs to be dumped into landfills despite various techniques available to manage different types of waste [18,22]. The final destination of garbage is the landfill, where before monitoring and extraction, controls are in place to prevent environmental impacts such as soil contamination through leachate releases or greenhouse gas emissions from methane [23]. Landfills, in addition to taking up large amounts of space, have been known to encroach on natural habitats. Such as highlighting the risk from spontaneous combustion of methane that worsened overall air quality and public health due to a complex mixture of toxic fumes [10] e.g. the recent Ghazipur landfill fire in Delhi was a common hazard [24]. Converting landfill waste to RDF can convert indispensable residential waste into industrial furnaces and power plant fuels, removing these materials from the dump and supporting increased energy production [22].

## 3 Production of RDF

Although RDF has some promise, it is still challenging to mass produce in developing parts of the world. The main challenge is the variability of waste inputs. The quality of household waste, which is the main input for RDF, changes considerably depending on seasonal, regional and social conditions. This variation impinges on the calorific value and thus leads to the quality of RDF, which is difficult for achieve a uniform fuel that can be burnt constantly in industrial facilities, for example, cement kilns etc [25]. However, the biggest bottleneck continues to be maintaining fuel quality up to industry standards. A number of technical challenges obstructs the process of RDF production. It is also very energy-demanding, where the many stages, from shredding over-drying to separation, involve high operational costs. Moreover, if contaminated by plastics (polyvinyl chloride and polyethylene terephthalate), chlorinated materials, heavy metals, etc., it can produce toxic emissions containing dioxins and furans during combustion [26]. The segregation of RDF uses infrastructure such as shredders, dryers, and separation systems, costing considerable capital infrastructure costs vary depending on prevailing markets, making some overburdened for emerging markets. RDF is economically advantageous because of its revenues but stands less competitive against traditional fossil fuels when one looks at the expenditure cost of emission control systems and maintaining hazardous by-products such as ash [27]. Overall, the high variability of waste inputs, technical complexities, and economic costs make large-scale RDF production difficult for developing regions. Addressing these challenges is crucial to making RDF a scalable and sustainable energy solution, especially in regions with limited resources and infrastructure.

**Fig. 4:**
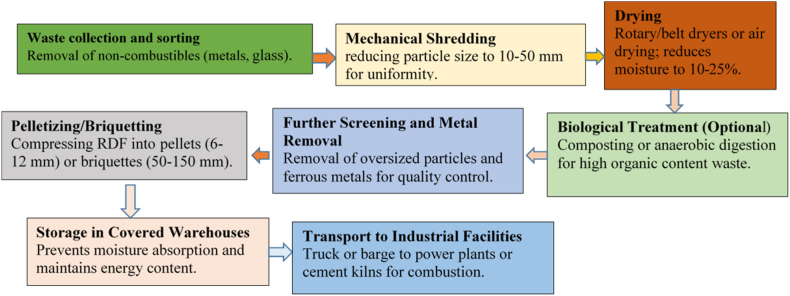
Steps to generate RDF from MSW and commercial sources.

The segregation of the RDF process helps to separate the waste, including non-combustible materials that will removed (and, where possible, recycled), e. g., metal elements, glasses, as well as other mineral fractions which can be reused in the RDF production phase (Fig. 4) [4,26]. Waste is washed and processed to the desired homogeneity of product particle size typical for slag-free feed waste into sizes >10 < 50 mm through mechanical means of shredding, etc. Drying — in the next step, RDF must be dried to a moisture content of 10–25 %, A higher value in this range reduces fuel quality and caloric value, which can result in low calorific values for the final fuel, typically between 15 and 25 MJ/kg, it all depends on waste composition [27]. RDF is primarily dried in rotary or belt dryers, although natural air drying is sometimes used. Most of the energy for drying is obtained from waste heat of industrial processes or power stations. The critical point in this sense is to attend an increase in RDF energy density that provides a decrease of transportation cost. For waste with high organic content, biological treatments in the form of either composting or anaerobic digestion can be employed, which is then followed by RDF production to reduce volumes as well as moisture contents of biodegradable wastes [[28], [29], [30]]. The material is then further processed after the initial shredding and drying, which include de-dusting it to remove heavy dust particles as well as oversized particles and ferrous metal contaminants through screening. It ensures that fuel stays the same at all times. This RDF is thereafter usually palletized or briquetted into higher density, pellet sizes typically range from 6 to 12 mm and breadths of between 50 and 150 mm [31, 32. This process compresses RDF, rendering it 0.6–0.8 g/cm³ of bulk density from lower ranging 0.15–0.3 g/cm³, making both storage and transport more efficient [33,34]. As a good quality alternative fuel for industrial use, the RDF has to fulfill appropriate specifications on calorific value, moisture content and chemical composition. Though high ash content, generally 10–25 % may lower the combustion efficiency; and higher levels (0.5–1%) of chlorine and sulfur pollutants must be dictated to avoid formation of hazardous emissions [21,28]. Therefore, environmental emission standards must be met by RDF before it is utilized given that it is an energy source. After the RDF is processed, it is kept in solar-covered warehouses to avoid absorbing moisture, as this would reduce its energy potential. Since RDF has an even higher bulk density than the currently loosely disposed-of waste, it can be transported at reasonable cost over longer distances of up to 1000 km by truck or barge. They are large power plants and cement kilns wherein energy production and disposal of municipal solid waste are combined to put the fuel value to its best use and reduce the environmental impact.

**Fig. 5:**
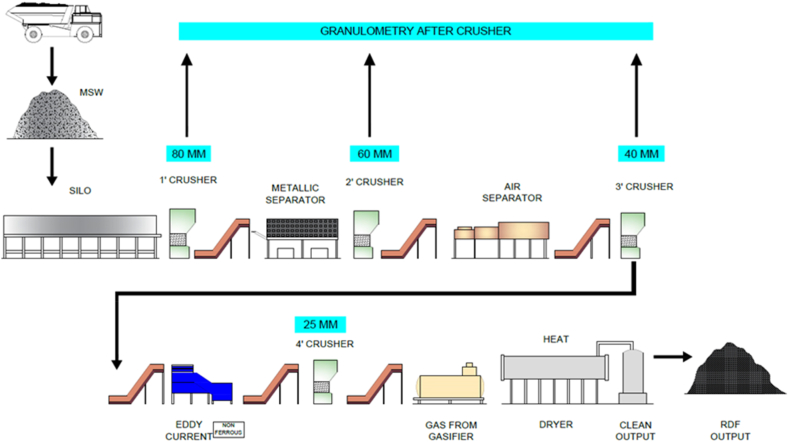
Pictorial view of separation of RDF using various machinery.

Fig. 4, Fig. 5 illustrates a streamlined process for converting MSW into RDF, showcasing significant improvements in plant efficiency and output quality over conventional systems. After dewatering, MSW is crushed progressively to sizes of 80 mm, 60 mm, and finally, 25 mm using a high-efficiency Pulverizer. Particle sizes are optimized to meet stringent criteria, resulting in high-quality RDF. Integrating advanced technologies, such as automated sorting systems, further boosts productivity. Magnetic and eddy current separation optimizes metal recovery, reduces the risk of contamination and enhances RDF quality. On the potential for upgrades, air separators eliminate lightweight plastics, and top-of-the-range sensors will achieve RDF higher purity through automatic detection of undesired materials. These upgrades not only endorse the high calorific value of RDF but also cut processing costs, leading to sustainable and profitable RDF production. Higher energy recovery rates and overall environmental performance of creating RDF thereby fit into global sustainability goals and seem to offer crushing, separation, drying, and emissions control advancements surveyed in the wider context of technological progress.

Production of RDF is known to have positive environmental and economic benefits but objectifies some challenges as well. From an ecological perspective, RDF helps avoid consigning large amounts of municipal solid waste to landfills as input into biodegradation and potentially methane release (a significant greenhouse gas (GHG) and resource extraction - both before and when downcycled – or contamination of resources (land, water) [26,28]. RDF helps reducing the greenhouse gases, by recycling of waste into circular economy and safe combustion. Furthermore, RDF is a cheap replacement for coal, natural gas and other fossil fuels which are widely used in cement kilns, power plants etc [35].

RDF, as a carbon-neutral renewable energy, can help reduce reliance on non-renewable sources. RDF combustion is cleaner than coal, but if waste with plastics or poisonous materials is incinerated, it can still emit toxic substances such as dioxins, heavy metals, and particles. However, implementing effective air filtration and emissions control systems will increase operational costs. Furthermore, RDF production facilities demand substantial capital investment, making them potentially prohibitively expensive for smaller sites. Another aspect of the environmental impact is that the combustion of RDF generates toxic ash, which can be detrimental to the environment if not managed properly. Although RDF is a promising green fuel, regulating its emissions and associated costs is crucial to maintaining its status as an environmentally responsible and cost-effective solution [32,34,35].

## 4 Classification of RDF

RDF classification is based on seven key factors determining its quality and application. Calorific value is vital, with high-quality RDF exceeding 15 MJ/kg, suitable for energy-demanding processes like cement kilns. In comparison, lower-grade RDF with values below 15 MJ/kg is used in waste-to-energy (WtE) plants [30]. Table 2 shows the parameters from which the quality of RDF was determined. Moisture content should remain under 20 % for optimal energy output and efficient combustion, as high moisture reduces calorific value [30,34]. Ash content of less than 10 % is preferred to reduce disposal costs and improve combustion efficiency. Particle size also matters, with smaller, uniform particles improving fuel feeding and combustion efficiency in industrial processes. Chemical composition is critical, especially chlorine, which should be kept below 1 % to minimize toxic emissions like dioxins and furans. Similarly, sulfur content should be low to avoid harmful sulfur dioxide emissions [32,36]. Contaminants like plastics and heavy metals negatively affect RDF quality, necessitating advanced pollution control measures when utilized in lower-grade fuels. The bulk density affects the cost of transportation and storage, where high-density RDF is of economic value for transporting to longer distances [20]. Classifying these factors could help find the right amount of trade-off between economic efficiency and environmental compliance.

**Table 2:** Various parameter to determine the quality of RDF [[36], [37], [38], [39]].

Parameter	RDF Specifications	RDF_Q (high quality) Specifications
Moisture	Maximum 25 % (as is)	Maximum 18 % (as is)
LHV	Minimum 15 MJ/kg (as is)	Minimum 20 MJ/kg (dry matter)
Ash content	Maximum 20 % (dry matter)	Maximum 15 % (dry matter)
Arsenic (As)	Maximum 9 mg/kg (dry matter)	Maximum 5 mg/kg (dry matter)
Cadmium (Cd)	No limit set	Maximum 3 mg/kg (dry matter)
Mercury (Hg)	No limit set	Maximum 1 mg/kg (dry matter)
Combined Cd + Hg	Maximum 7 mg/kg (dry matter)	No limit set
Total Chlorine (Cl)	Maximum 0.9 % (as is)	Maximum 0.7 % (dry matter)
Chromium (Cr)	Maximum 100 mg/kg (dry matter)	Maximum 70 mg/kg (dry matter)
Soluble Copper (Cu)	Maximum 300 mg/kg (dry matter)	Maximum 50 mg/kg (dry matter)
Manganese (Mn)	Maximum 400 mg/kg (dry matter)	Maximum 200 mg/kg (dry matter)
Nickel (Ni)	Maximum 40 mg/kg (dry matter)	Maximum 30 mg/kg (dry matter)
Volatile Lead (Pb)	Maximum 200 mg/kg (dry matter)	Maximum 100 mg/kg (dry matter)
Sulfur (S)	Maximum 0.6 % (as is)	Maximum 0.3 % (dry matter)
Glass content, Fluorine, Aluminum, Tin, Zinc, Exterior aspect, Dimensions, Ash softening temperature	No limit set	No limit set

**Table 3:** Comparative analysis of RDF composition across various studies.

Component	Study 1 [6]	Study 2 [22]	Study 3 [26]	Study 4 [27]	Study 5 [28]	Study 6 [30]	Study 7 [37]	Study 8 [47]	Study 9 [35]
Plastics	3.6 %	19.1 %	22 %	8 %	42.1 %	19.7 %	8 %	36 %	8.8 %
Rubber	–	–	–	–	–	–	6 %	–	–
Textile	66 %	2.9 %	20 %	9.9 %	7 %	21.3 %	6 %	6 %	22.2 %
Paper/Cardboard	17.1 %	26 %	10 %	22.2 %	41.2 %	3.4 %	20 %	23 %	12.4 %
Composites/Synthetics	–	–	5 %	–	–	–	–	–	8.8 %
Glass	–	–	–	–	–	–	–	–	1.4 %
Metals	–	–	–	–	–	1.8 %	–	–	5.2 %
Organic	–	10 %	–	14.3 %	9.6 %	–	29 %	–	35.2 %
Inerts	–	–	–	4 %	–	–	–	–	–
Plastic Film/Bags	13.3 %	–	–	33.2 %	–	21.8 %	–	–	–
Unidentified	–	34.5 %	20 %	6.8 %	–	4.9 %	20 %	35 %	2.9 %
Fine Particles	–	1.3 %	22 %	1.6 %	–	10.4 %	–	–	3 %
Wood/Leather	–	6.2 %	1 %	–	–	16.7 %	11 %	–	–
Calorific Value (kJ/kg)	3988.8	3773.8	–	6401.2	5660.7	2555.7–4896.4	5923.5	–	2221.3

Despite its longstanding presence, the RDF industry has experienced numerous challenges, primarily due to inconsistent fuel quality and skepticism towards the reliability of waste-derived fuels [40]. As a result, between 1990 and 2010, several European countries introduced national standards [41]. These standards were designed to establish numerical guidelines enabling producers to generate RDF with reliably predictable quality [28]. These standards classify RDF into two groups based on specific technical criteria, including Net Calorific Value (NCV), moisture content, ash content, and the presence of pollutants such as chlorine and mercury [38]. These documents are descriptions of what RDF and Quality Controlled RDF (RDF_Q) are about and how it is defined and legally designed, including things such as definitions, sampling methodologies, critical parameters and evaluation methods needed for securing the fuel consistency, homogeneity and safety (refer Table 2). The first RDF classification system to be known was pioneered by the American Society for Testing and Materials (ASTM) [42]. This system involved a 7-class structure to classify RDF according to qualitative descriptors.

The categories ranged from raw waste (RDF-1) and treatment fuel (RDF-5) to gaseous fuel produced by the waste incineration process (RDF-7) [42]. There are seven categories of RDF, depending on the waste material's preparation level and physical form. From waste in its raw form to almost refined gaseous fuels, these classifications can have a wide range of uses across several industries. Type RDF-1 is waste that has been sorted but left in its predominantly heaped, unprocessed form and contains all organic materials from MSW and some non-combustibles. RDF-2: Producing Course RDF (Shredded without separation of ferrous metals): The second type consists of shredding the waste to reduce it into coarse particles, which helps in easy handling and combustion (even with or without separating 20–30 mm). RDF-3, which is better separated via non-combustible material like metals, with glass and irregular inorganic substances, is removed as well before the waste is shredded to a more uniform size for combustion. RDF-4 takes the waste collected and processes it into a powdery form, making it easier to burn. It converts the waste into pellets or briquettes to store and transport it more efficiently, improving combustion efficiency. This densified RDF is used in cement kilns, industrial boilers and combined heat and power (CHP) centralized plants. Taking RDF-6, waste can be further processed into liquid fuel by a series of advanced processes, which allows it to be used in combustion engines and other systems based on liquid fuel. RDF-7 is produced as gaseous fuel through gasification or pyrolysis processes, and thus, the most advanced RDF suitable for gas turbines [38,40].

Both national and international standards guarantee quality, safety, and environmental compliance in the production and use of RDF. One significant regulatory body for the classification of RDF is the European Committee for Standardization (CEN) through CEN/TC 343 [40]. One of the most significant specifications is CEN/TS 15359, which ranks RDF according to its NCV, chlorine, and mercury content [40]. The RDF quality framework set essential energy and environmental standards for applications in the industries such as cement and power plants. Furthermore, metal concentrations regulated by CEN standards are also controlled to reduce risks of health and environmental problems during combustion. Outside the E.U., RDF production can be governed by the International Organization for Standardization (ISO), a global body such as ISO 21640, which is an ISO standard that specifies methods for sampling, testing, and analysis of solid recovered fuels [33]. By design, this will ensure the RDF produced meets international quality and safety standards common for export trade, enabling RDF to cross country barriers in an environmentally sustainable process.

The management of RDF is regulated by the European classification system and the BS EN 15359 standard in the U.K. Standards related to RDF can be referenced in Chavando et al. [40] additional regulations have been implemented to promote the transport of thermally treatable waste to WtE facilities. In India, RDF production is overseen by the Ministry of Environment, Forest and Climate Change (MoEFCC) under the Solid Waste Management Rules, 2016 [28]. These rules outline the sourcing, processing, and characterization of hazardous materials. Certifications like EN 15359 and ISO are essential for ensuring the quality of RDF for industrial applications [42]. Proper raw material composition not only guarantees suitability but also ensures compliance with stringent safety regulations, particularly for industries such as cement kilns.

## 5 Case study: detailed analysis of RDF from five different locations in Uttar Pradesh India and their comparison

**Fig. 6:**
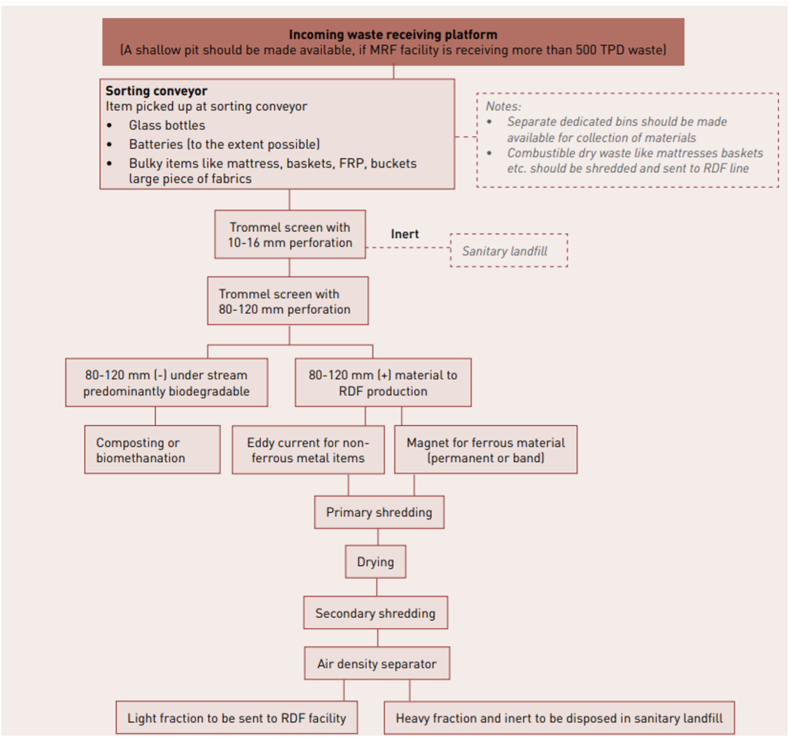
Flow chart for the pre-processing of mixed municipal waste [3].

In Uttar Pradesh and other state of MSW sites in India, the segregation of RDF and organic waste is carried out mechanically, as shown in the flowchart of Fig. 6. The flowchart represents the feedstock treatment and waste management processes of a Material Recovery Facility (MRF) capable of handling both RDF production and organic matter separation in all the state of India.

Waste initially enters the *Incoming Waste Receiving Platform*, where a shallow pit is used for processing at facilities handling at least 100 tons per day (TPD). Separate bins should be provided for different materials, and any combustible dry waste items, such as mattresses or baskets, must be shredded and routed to the RDF line. The waste then moves to a *Sorting Conveyor*, where specific items, such as glass bottles, batteries, and bulky items like mattresses, baskets, fiber-reinforced plastic (FRP), buckets, and large pieces of fabric, are manually removed. The remaining waste undergoes *Trommel Screening*, starting with a trommel screen with 10–16 mm perforations to sieve out fine inert materials, which are sent to a landfill. From here, the waste moves to a second trommel screen with 80–120 mm perforations, which further separates it into two streams. Stream 1 consists primarily of biodegradable under-sized particles (80–120 mm), which are sent for composting or biomethantion. Stream 2, which contains coarser material suitable for RDF production, goes through additional processing.

The RDF-bound material undergoes *Eddy Current Separation* to remove non-ferrous metals and *Magnetic Separation* to recover ferrous materials using either permanent or band magnets. After sorting, this RDF material undergoes *Primary Shredding*, followed by drying and *Secondary Shredding* to achieve the desired particle size. The RDF is further refined using an *Air Density Separator*, where it is divided into a light fraction that is sent to the RDF facility and a heavy fraction (including residual inert materials) that is disposed of in a sanitary landfill. This entire process is designed to maximize resource recovery, optimize RDF production, and minimize landfill disposal.

**Fig. 7:**
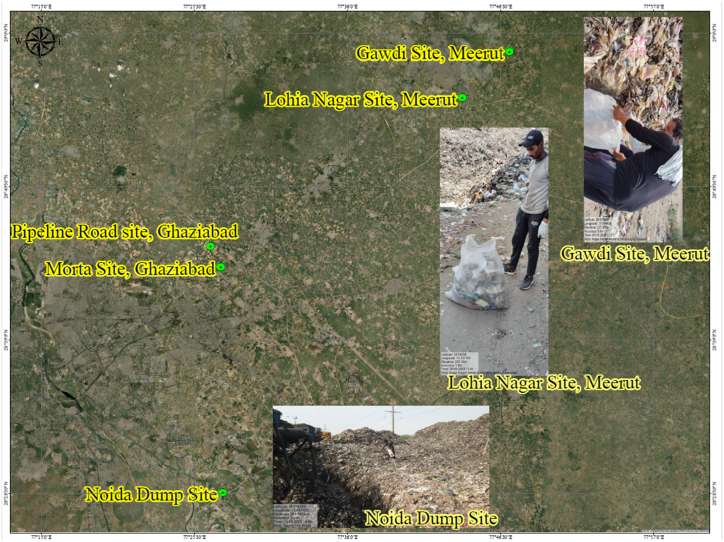
Geographical map illustrating the location of the test site.

RDF samples were collected from five strategic locations in Uttar Pradesh: Morta Site (28.7294° N, 77.4917° E), Pipeline Site (28.7104° N, 77.4627° E), Sector 146 Noida (28.5443° N, 77.3863° E), Lohia Nagar (28.6724° N, 77.4328° E), and Gawdee (28.6938° N, 77.5044° E). These sites, chosen for their diverse urban and semi-urban waste sources and proximity to industrial areas, provide a range of waste types. Morta and Noida sites offer relatively fresh waste, while Pipeline and Lohia Nagar include older, partially degraded waste. Gawdee receives mixed residential and agricultural waste, showcasing varied decomposition levels. The RDF sample was collected following the separation of RDF from MSW, as illustrated in Fig. 7. In Uttar Pradesh, RDF bio-mining is conducted using the same process shown in the flowchart. At the older site, RDF was collected after separation during the bio-mining process. A 4 × 4 feet area was designated for segregated RDF according to the flow chart for sample collection. Samples were extracted from three distinct layers: top, middle, and bottom.

The size of the area for segregated RDF sampling was selected as 4 × 4 feet to provide a standard sample size that, considering the heterogeneity of the RDF material, ensures a representative sample. This dimension facilitates the extraction of an adequate amount of RDF from a defined area, encompassing multiple profiles (top, middle, and bottom). Following the protocols outlined in the bio-mining flow chart (Fig. 7), this method was used to maintain consistency in sampling across sites. The defined area allowed for the capture of varying RDF properties based on waste type, decomposition stage, and differences between sites.

The samples were collected from five locations within each site and subsequently homogenized to create a representative RDF sample. Extensive testing was performed on the RDF samples from these sites to evaluate their physical characteristics, chemical composition, and heavy metal content. Physical characteristics evaluated included moisture content, ash content, volatile Matter, fixed Carbon, and bulk density, as shown in Table 4. Chemical analyses focused on total organic Carbon, total Nitrogen, volatile compounds, calorific values (gross and net), and total chloride content are shown in Table 5. Additionally, heavy metal testing was performed to quantify concentrations of lead (Pb), nickel (Ni), arsenic (As), zinc (Zn), cadmium (Cd), chromium (Cr), copper (Cu), mercury (Hg), and other trace metals is shown in Table 6. The results from these analyses provided a detailed characterization of the RDF samples across the various collection sites.

**Table 4:** Physical characteristics of RDF.

Parameter	Morta Site	Pipeline Site	Sector 146 Noida	Lohia Nagar	Gawdee
Moisture Content (%) by mass	15.22	8.22	8.20	20	18.30
Biodegradable Quantity (%) by mass	28.5	25.2	30.1	18.5	23.1
Non-Biodegradable Quantity (%)	71.5	74.5	69.2	80.5	85.8
Domestic Hazardous Waste (%)	2.8	2.2	1.08	1.05	0.98
Ash Content (%)	56.80	55.80	52.80	51.80	52.80
Volatile Matter (%)	28.37	35.37	40.37	30.07	28.37
Fixed Carbon (%)	4.51	4.81	3.11	6.11	3.01
Conductivity	3880	3650	3050	3930	3100
pH	9.03	7.86	8.04	9.10	9.05
Bulk Density	356	338	316	376	366

**Table 5:** Chemical characteristics of RDF from different sites.

Parameter	Morta Site	Pipeline Site	Sector 146 Noida	Lohia Nagar	Gawdee
Total Organic Carbon (%)	28.37	0.48	0.37	28.37	0.57
Total Nitrogen (%)	0.22	0.22	0.017	0.22	0.22
Volatile Compounds (%)	15.20	10.20	8.20	15.20	18.20
Gross Calorific Value (kJ/kg)	1680	1680	1250	1510	1540
Net Calorific Value (kJ/kg)	1450	1450	1120	1420	1410
Total Chloride (%)	0.046	0.046	0.025	0.036	0.035
Total Hydrogen (%)	3.12	2.71	2.20	3.00	3.25
Total Oxygen (%)	21.00	25.00	26.50	20.80	20.00
Other Elements^a^ (%)	32.04	61.32	62.67	32.35	57.68
Total (%)	100.00	100.00	100.00	100.00	100.00

a Other Elements-include materials such as ash, inert materials, metals, and other trace elements that are not explicitly measured in this dataset but are typically found in RDF or similar waste materials.

**Table 6:** Heavy metal characteristics of RDF from different sites.

Parameter (mg/kg)	Morta Site	Pipeline Site	Sector 146 Noida	Lohia Nagar	Gawdee
Lead (Pb)	28.40	27.20	22.70	20.10	30.10
Nickel (Ni)	6.40	6.50	3.70	5.40	7.10
Arsenic (As)	5.02	5.02	6.02	3.02	5.55
Zinc (Zn)	22.10	25.10	29.15	29.00	12.12
Cadmium (Cd)	0.36	0.36	0.26	0.36	0.39
Total Chromium (Cr)	12.10	12.10	10.52	4.10	2.10
Copper (Cu)	4.60	0.60	0.60	2.60	0.60
Mercury (Hg)	<0.01	<0.01	<0.01	<0.01	<0.01

The composition of RDF significantly depends on the type of waste and the pre-processing methods used. Understanding these compositions is crucial for optimizing RDF production and enhancing its effectiveness as a fuel source. Data in Table 3 indicate that RDF compositions vary, reflecting different collection sites, seasonal factors, and processing methods, making it difficult to establish a benchmark for waste composition. Older waste typically has a higher decay rate, leading to variable calorific values. For instance, plastic content ranges from 3.6 % in Study 1–42.1 % in Study 4, contributing to Study 4's higher calorific value (6401.2 kJ/kg) compared to Study 1 (3988.8 kJ/kg). Textiles and paper content also influence energy output, with higher textile ratios resulting in lower calorific values. Moreover, the presence of organic materials and inerts, such as metals and glass, can further diminish RDF's energy potential, underscoring the importance of effective waste sorting and processing for high-quality RDF.

Several steps are involved in the proximate analysis process to determine the composition of RDF and some particular characteristics. ASTM standard D3173 is used to determine RDF's moisture content as a (1 g) sample in an uncovered crucible is placed and heated at 105 °C in a muffle Furnace for 1 h. The sample is cooled and reweighed, then the moisture content is calculated. Then, for calculating the volatile matter content under ASTM standard E872-82, a dried sample is rinsed with xylene, covered and heated in a 950 °C furnace for 7 min. The sample is then cooled in a desiccator and weighed to determine the weight loss through volatilization. The ash content is determined by heating the residual sample in an oven to 500 °C for 2 h per ASTM standard D1102. The ash content is determined by weighing the scorched remains. Lastly, fixed carbon percentage is obtained by deducting total weight from the sum of moisture %age + volatile matter %age + ash %age. Bomb calorimeter (Digital Beckmann, Abacus Instrument) determines the Calorific value, i.e. Higher Heating Value (HHV) or gross calorific value of RDF samples. This method consists of burning a mass of the RDF sample inside the bomb calorimeter and measuring the temperature variation (ΔT) of water around it. This calculation requires the water's specific heat (Cp) and mass (MW). Next, the HHV is worked out by multiplying the mass of water through its particular heat and rise in temperature, and afterwards, this value is divided by the mass of the RDF sample. The clarity in which the method measures the energy content of the RDF is fundamental to screening it as a fuel source. Identification of suitable quality and composition based on the physical, chemical, and heavy metal characteristics was done for industries viz. cement manufacturing, power generation plant and waste-to-energy plants to use specific RDF samples. This will, in turn, give us perspective on how RDF use can be optimized for environmental sustainability and resource efficiency while ensuring environmental project compliance and economic commercial viability.

The present study investigates the calorific values and fuel quality of RDF samples collected from five different locations in Uttar Pradesh, hinting at the extent of variability in the fuel characteristics across locations. Of the five sites, Morta and Pipeline Site have a maximum GCV of 1680 kJ/kg each, indicating these are locations producing high-quality RDF. Features such as mixed industrial data and domestic waste, low moisture content, but high calorific are the basis of high caloric values in these sites. On the other hand, Sector 146 Noida is also found to have the lowest GCV of 1250 kJ/kg, which can be attributed to the high moisture content and nature of waste generated in densely populated urban areas. However, the calorific values of RDF based on this study were within the lower range of calorific value compared to those reported by other studies conducted in similar regions. RDF calorific values have been recorded between 3000 and 4500 kJ/kg, but the higher ones are frequently in areas that are decreasingly more urbanized or practising improved waste segregation. This implies a high scope of enhancing the RDF production in Uttar Pradesh with better waste treatment and segregation methods. Feedstock composition is the primary factor that determines the quality of RDF. The more non-biodegradable content (like plastics and textiles) there is, the more calorific the value of RDF. Lohia Nagar (80.5 %) and Gawdee (85.8 %) show a high percentage of non-biodegradables. However, they are still in a moderately clarified material, which indicates that the former two parameters greatly determine the quality of RDF, not just raw waste components. The moisture content, which is mainly responsible for calorific value, has also been studied. Sector 146 Noida and Pipeline Site also show a moisture level of 8 %, which is lower than that of the other sites. Nevertheless, despite their low moisture content, occasions only achieve moderate calorific values at these sites, suggesting a dependence of fuel quality on waste composition. This, coupled with high artefactual waste such as industrial waste (which is not calorific and will not contribute as much to the CV as plastics or textiles), could contribute to these results. This shows that a decrease in moisture content alone does not assure higher calorific values; the quality of waste also influences the same. Morta and Pipeline Sites had the highest RDF heating values, illustrating the role of waste composition on fuel quality. The fact that these sites receive a combination of low moisture content and a blend of industrial and household waste helps improve their quality RDF. In conclusion, the study highlights that RDF properties are not uniform throughout different sites because FCV and MC mainly determine fuel characteristics. This variable emphasizes the need for location-specific approaches to increase RDF production.

### 5.1 TGA experiment to assess the viability of RDF samples

**Fig. 8:**
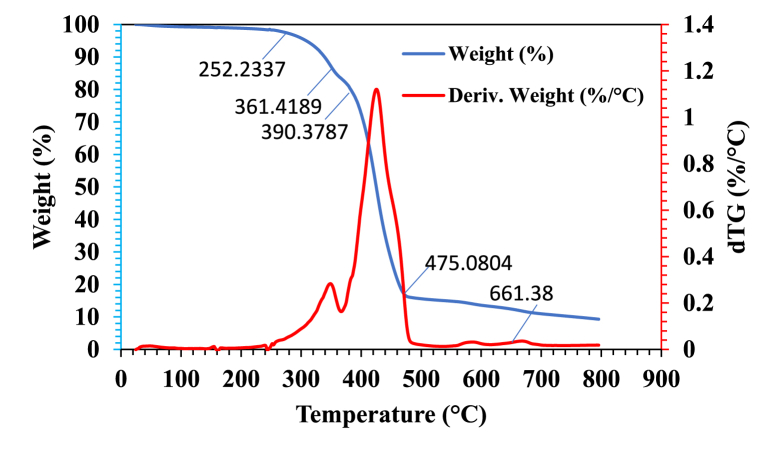
TGA and dTG analysis of RDF.

The TGA presented in Fig. 8 shows the thermogram of the RDF sample, determining its feasibility level. Before testing, we pooled the samples from five sites to represent the among-site variation in composite samples. The thermal decomposition of the RDF sample was analyzed using Thermogravimetric analysis (TGA) and derivative thermogravimetric analysis (dTG). TGA can track the temperature-dependent weight losses to estimate the inherent velocity of microbial growth. At the same time, the DTG curve guides towards specific phase transitions in the decay behaviours of the RDF sample. These findings will benefit the safe use of RDF in various industrial applications by obtaining a good insight into its thermal stability and degradation behaviour. The results provide information on the degree and timing of decomposition at different temperatures, including plastics, biomass breakdown (ecological impacts), and other components in RDF, which have implications for assessing its suitability for gasification or thermal treatment by pyrolysis. The testing was performed using a second-generation simultaneous TGA/DSC instrument (SDT Q600) from D.E., USA. This advanced equipment allows for simultaneous analysis of two samples, ensuring consistency and reproducibility of results. The instrument records weight changes along with heat flow, providing complementary data on the thermal behavior of the RDF. The insights gained from these analyses have implications for the safe and efficient utilization of RDF in various industrial applications, offering a better understanding of its thermal stability, degradation stages, and environmental impacts.

The 9.865 mg samples were placed in standard alumina sample/reference holders and heated at 10 °C/min from 30 to 900 °C under a continuous purge of purified Nitrogen (N_2_). The flow of Nitrogen was adjusted to 100 mL/min, and that is because we want to prevent any possible afterburning or secondary reactions within the gas-fuel mixture so the environment will be inert. The noise signal from the empty crucible was captured and subsequently subtracted from signals during the experiments. Calculations were made to determine the actual mass of the sample based on the correlation of weight loss with temperature.

A thermogravimetric analysis was first used to investigate the thermal breakdown and find an appropriate temperature range for pyrolysis, as shown in Fig. 8. Results showed that pyrolysis started at temperatures higher than 250 °C, and in the nitrogen atmosphere, the three following separate phases were recognized during the thermal degradation of the RDF sample. The first phase recorded slight weight loss (probably moisture in the sample). The decomposition stage was followed by the next phase, in which primary components were broken down as a significant weight loss occurred. Then, a slight weight loss was detected, probably due to the degradation of carbonaceous materials. Since the sample had major HDPE and LDPE contents (71%–80 %) with the addition of biomass (13%–20 % - paper, cardboard). The TGA measurements and derivative thermogravimetric analysis dTG provided information as follows:

The analysis indicates that the maximum fire temperature is unlikely to exceed 250 °C, despite evidence that low-density polyethylene (LDPE) combusts in air at temperatures exceeding 200 °C. The derivative thermogravimetry (DTG) peak at 355 °C suggests initial degradation processes, while the subsequent peaks at 361 °C and 390 °C likely correspond to the further breakdown of specific plastic constituents, with each peak indicating the decomposition of different plastic types at their respective degradation temperatures. The DTG peak at 435 °C effectively distinguishes the pyrolysis of lignin, a significant biomass component derived from paper and cardboard. Given the minimal mass contribution of biomass during this phase, the weight loss observed is relatively low. Finally, the peak at 661 °C represents the gradual degradation of residual lignin, indicating the concluding stages of biomass pyrolysis. This peak may also signal the end of plastic decomposition. The TGA results reveal that the RDF sample experiences approximately 4 wt% mass loss up to 350 °C, establishing this temperature as a suitable maximum pyrolysis upper limit of 550 °C.

## 6 Utilizations of RDF

De Caevel et al. [43], identified several key drivers motivating industries to adopt RDF, emphasising the increasing cost of hydrocarbons as a significant factor. This escalation necessitates research into alternative energy solutions. Additionally, RDF is inexpensive and readily available; its disposal can often be cheaper than landfill options, especially in light of bans and taxes. RDF can be utilized in various waste-to-energy processes, including combustion (in burn incineration plants or dedicated waste-to-energy facilities), gasification, and pyrolysis. These methods leverage RDF to convert waste into energy, promoting more efficient management of municipal and industrial waste and reducing reliance on traditional fossil fuels. RDF gasification converts waste into gaseous fuel in the presence of oxygen, leading to a solid by-product with a carbonaceous compound [44]. To this end, over 100 waste gasification plants were in operation worldwide by 2002 [45], with higher quality requirements making RDF well-suited for such applications.

Pyrolysis is a new advanced technique that occurs without oxygen but produces higher-calorific-value gas. This process is temperature-flexible and typically part of a more integrated facility. It combines with gasification to maximize efficiency between the two smaller processes running at optimal temperatures for their respective technologies. However, significant energy is consumed to produce RDF, which substantially reduces the overall energy potential of this technique [46]. Still, high and consistent quality offers benefits such as compatibility with all reactor types, leading to steady operation and flow distribution uniformity.

### 6.1 Utilization of RDF in cement industry

**Fig. 9:**
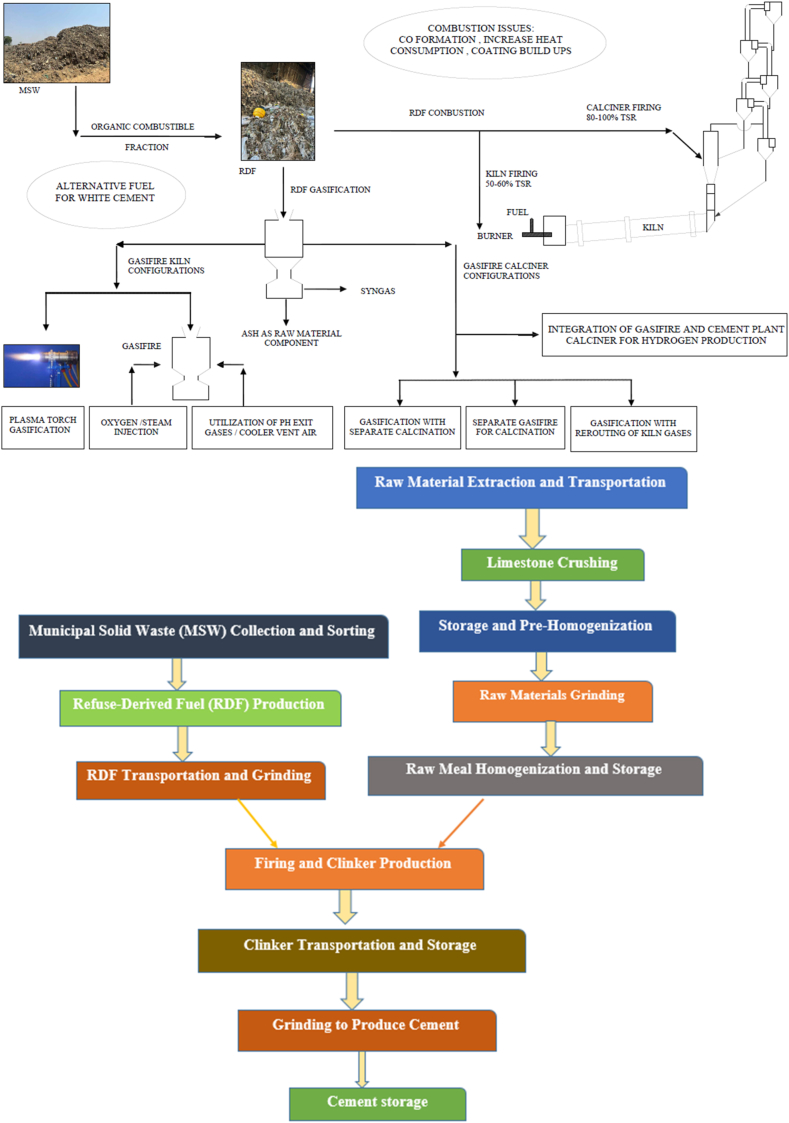
Utilization of RDF in cement industry [29,30,35,37].

Fig. 9 shows the schematic representation of utilization of RDF in the cement industry. Cement kilns are one of the fastest-growing RDF consumers as they have switched to using supplementary fuel instead of traditional fossil fuels such as coal and petroleum coke [42]. The concept of incinerating RDF is also particularly well-suited to be carried out in cement kilns (which typically operate at temperatures significantly above 1,400 °C) because the high temperature ensures complete combustion of even difficult-to-burn fractions, giving much more residential time for Poisonous organic substances as whilst kindles some moderate and assists with clinker delivery [9,30]. The dual functionality of RDF as both a feedstock for fuel production and a waste management solution makes it a compelling option. High-temperature kilns effectively combust all organic and inorganic components of RDF, minimising residual ash that contributes to landfill volumes [14,28]. From a sustainability standpoint, RDF presents a more environmentally friendly alternative to fossil fuels, reducing carbon emissions. Cement plants utilizing RDF can decrease their CO₂ emissions by 30 % compared to those that rely on coal [47]. Some of this reduction comes from the lower carbon intensity of RDF compared to incineration. However, it also arises because we are diverting waste from landfills (and reducing their methane emissions). Further, RDF adoption carries with it economic rewards as well because RDF serves as a cheaper fuel option, which potentially will result in savings for cement manufacturers on account of a reduction in the cost of expensive traditional fuels while environmental credits or incentives linked to emission reductions [48].

Refuse-derived fuel used in cement kilns has a mean calorific value of 15–25 MJ/kg, which is essential for the clinker production process due to technical constraints [35]. For efficient feeding and combustion, RDF must be finely ground; coarser particles can lead to uneven operations and increased emissions [37,48]. Complete combustion of RDF in cement kilns often requires improved airflow systems and advanced combustion monitoring methods. The primary afterburner system incorporates internal secondary combustion, which reduces approximately half of the NOx emissions and nearly all CO emissions through a two-stage process before adding additional fuel [41,42]. This process also aids in oxidizing potassium minerals and removing other contaminants, such as dioxins and mercury. If RDF is combined with mineral fillers, additional emission control systems, such as scrubbers or aqueous lime treatments, may be necessary [47,48].

To optimize RDF combustion while meeting environmental performance standards, operators must adjust oxygen levels and flame temperatures. Emission and fuel quality standards apply to RDF fed into cement kilns, with European guidelines setting limits on calorific value, chlorine content, and heavy metal inputs [30,35]. In India, the MoEFCC has established emission standards for pollutants like NOx, SO₂, and particulate matter, requiring Continuous Emissions Monitoring Systems (CEMS) for real-time measurement [28,42]. Although these regulations present challenges, achieving emission targets consistently relies on performance-based testing methods applied to lower-grade RDF. Consequently, many cement plants implement advanced emissions control strategies, such as Selective Catalytic Reduction and Wet Scrubbers, to ensure compliance [10,14,34].

Countries like Germany, Italy, and Sweden have led the integration of RDF into European cement production. Germany's cement industry consumes around 1.5 million tons of RDF annually, significantly reducing waste and recovering energy [38,41]. Similarly, Italy's cement manufacturers, including Italcementi and Buzzi Unicem, extensively use RDF to leverage its high calorific value, maintaining efficient kiln temperatures, as shown in Table 8. Table 9 summarises the application of RDF in various cement industries in 2024, based on the latest verified data from Global Cement.

**Table 7:** Broad guideline defining parameters of raw MSW and RDF in India [49].

Parameter	MSW	RDF 1	RDF 2
Size	<400 mm	<75 mm	<35 mm
GCV	>1500 Cal/gm	2500–3500 Cal/gm	2500–3500 Cal/gm
Moisture	No limit	<25 %	<25 %
Ash	No limit	<20 %	<20 %
Chlorine	No limit	<1 %	<1 %

**Table 8:** Quality requirements for RDF in cement power plants.

Parameter	Unit	Quality Requirement (RAL GZ 724) [51,52]
Net calorific value (NCV)	MJ/kg	13
Chlorine (Cl)	% in mass	0.7
Mercury (Hg)	mg/MJ	0.038

**Table 9:** Quality requirements for RDF in lime kilns.

Parameter	Unit	Quality Requirement [[51], [52], [53]]
Net Calorific Value (NCV)	MJ/kg	23
Chlorine (Cl)	% in mass	1
Mercury (Hg)	mg/MJ	0.022

In India, the adoption of RDF in the cement industry is progressively increasing, driven by government initiatives and stringent environmental regulations [1,5]. Leading companies such as ACC, Ambuja Cements, and Dalmia Bharat are at the forefront of integrating RDF into their fuel mix. The ACC Cement Plant has adopted RDF as part of its commitment to reducing its carbon footprint and landfill contributions. By using RDF, the plant has managed to substitute up to 30 % of its conventional fossil fuel consumption, saving on fuel costs and benefiting from the reduced emissions of RDF combustion [5,10,28]. However, the plant faced initial challenges with inconsistent RDF quality and high moisture content, which required modifications to the kiln feeding and combustion systems. These challenges were mitigated through improved RDF procurement practices and investments in drying technologies [38,42]. The broad guideline defining parameters of raw MSW and RDF in India is shown in Table 7.

Modern RDF is still a hit-and-miss proposition in India's cement sector. Many potential advantages remain to be gained by using better quality RDF for making clinker, but this nevertheless presents numerous challenges. RDF On the minus side, RDF is a critical player in landfill diversion and annually prevents 10 million tonnes of waste from going to landfills (Germany) — thus also saving GHGs. Another positive point of carboferric materials is that its calorific values can reach up to 15–20 MJ/kg, aiding the energy efficiency in cement kilns [41,49]. The co-firing of RDF and conventional fuels also results in a decrease CO_2_ emissions with additional benefits to the reduction of methane coming from landfills [50]. From a material and resource efficiency point of view, RDF combustion can be advantageous because the ash it produces might partially substitute for cement clinker (Puertas et al., 2008). The use of RDF may also create general economic benefits, such as 20 % fuel savings [9,29], more recyclable materials, or even turning waste into a well-suited raw material for the circular economy [11,35,39]. There are nevertheless many problems with the adoption of RDF. Materials and quality control performances also depend on variable storage characteristics and the nature of the bulk store structures [41,49]. Modifications of existing cement kilns will be very costly compared to other modifications (i.e., feeding system or combustion chamber and emissions controls) since it is a technical casing that depends on capital-intensive efforts [9,49]. The conceivable opposition to RDF application may relate to the possibility that its use will tend to reverse, as well as emission problems (NOx, SOx, and dioxins), necessitating enhanced control methods, including stringent discharge limit values [50]. Gaining public perception and acceptance when dealing with C&D waste over pollution and health issues could be challenging, eventually leading to resistance; therefore, clear communication among stakeholders is necessary 11, 25, 29]. Moreover, the rigid qualifying criteria set for using alternative fuels have led to difficulties and time-consuming procedures while applying for permits [26,41].

### 6.2 Utilization of RDF in coal power plants

Increasingly, RDF has been used as a fuel for power generation – notably in WtE facilities. RDF replaces conventional fossil fuels (i.e., coal and natural gas) by creating energy from unrecyclable waste. The fact that RDF serves a dual purpose—attending to waste management challenges and producing renewable energy—makes it extremely attractive for both power producers and garbage managers. RDF is also ideally suited to Combined Heat and Power (C.H.P.) units since these systems convert 70 % or more of the energy from fuel into electricity and proper heat [14,34,42]. RDF can indeed have a high impact on the energy side due to production levels: In Europe, RDF accounts for more than 10 TWh of power generation a year, delivered by an average plant capacity of around 40–60 MW electricity [14,24,38]. In countries with restricted landfill space, RDF, in a significant manner, ensures that up to 80 % of the amount of waste is sent out for final disposal. Only in the UK has the use of RDF resulted in landfills saving 15 % over the last decade [30,37,41].

• Calorific Value: RDF used for power generation should have a calorific value of 15–25 MJ/kg to ensure optimal operational temperatures to maximize energy production. Whilst this calorific value is lower than traditional fossil fuels like coal, on average 25–30 MJ/kg, RDF still produces a decent amount of energy when burned [28,34].
• Moisture content- Higher moisture content in RDF decreases combustion efficiency, reducing energy output and risking incomplete combustion. Optimal moisture content for RDF combustion typically ranges from 10 to 20 %; exceeding this can lead to operational inefficiencies and increased emissions [29,37].
• Components and Particle Size – RDF consists of varied materials like plastics, textiles, and paper. Particle size should be controlled for even boiler feed, typically between 30 and 300 mm, depending on combustion technology. Fluidized bed boilers require smaller particles for complete combustion, often achieved using a hammer mill [30,41].
• Ash Content – RDF combustion yields 10–30 % ash, higher than coal's average 5–10 %. Elevated ash levels necessitate efficient ash-handling systems and may increase maintenance costs [35,42].

RDF can significantly enhance plant efficiency. While a plant utilizing RDF may exhibit 5–10 % lower thermal efficiency compared to burning fossil fuels due to RDF's lower energy content, it is still considered one of the least environmentally polluting residual fuels [38]. This is largely because modern scrubbers and electrostatic precipitators are highly effective at capturing solid sulfur compounds, preventing them from being released into the flue gases. Additionally, RDF combustion tends to produce slightly higher emissions, but advanced capture mechanisms are more efficient in controlling NOx, SO₂, and particulate matter emissions from motor systems [46,49]. These systems improve the kinetics of amalgamation and enhance decomposition during continuous operations, demonstrating significant advancements in emissions control technologies [50].

A tonne of RDF offers significant cost advantages for power generation, with an estimated baseline price ranging from 20 to 40 euros per ton, which is considerably lower than coal prices, typically between €60 and €100 per ton [11,17,21]. This price disparity highlights the economic and environmental benefits of using RDF instead of landfills. Government subsidies, such as Germany's renewable energy credits, further enhance the profitability of RDF. Although retrofitting for RDF can require substantial investment, financing options like green bonds and carbon credits through the EU Emissions Trading System help make RDF initiatives more economically viable [11,27,31].

Refuse-derived fuel presents a cost-effective alternative to coal and natural gas, as it typically has a lower gate fee and tipping fees. With costs in the lower quartile at €20–€50 per MWh, RDF is an attractive option [28,29]. When burned, RDF produces 30–40 % less CO₂ emissions compared to coal and can reduce methane emissions in landfills by up to 80 %. Operationally, RDF is highly flexible, but its emissions must be carefully managed [38,47]. Utilizing RDF for power generation offers a scalable and energy-efficient advantage that surpasses the power output and emission reduction capabilities of technologies like incineration. Co-firing RDF with coal can cut CO₂ emissions by 30 %, thereby reducing environmental impact and enhancing energy security [27,31]. The diverse composition of RDF, including its ash content and varied feedstock types, can lead to restrictions on the quantity and type of materials accepted. Some facilities may heavily rely on emissions controls to meet compliance standards, posing additional challenges. In contrast, traditional fuels like coal and natural gas offer more consistent combustion but contribute significantly higher carbon emissions, adding a broader environmental impact.

RDF-based power generation also runs more energy efficient and is capable of scaling larger than most other waste to energy technologies, such as incineration or landfill gas recovery. Incineration plants mainly target degree of waste volume decline – RDF is capable of create not trifling quantities electricity with low emissions.

**Fig. 10:**
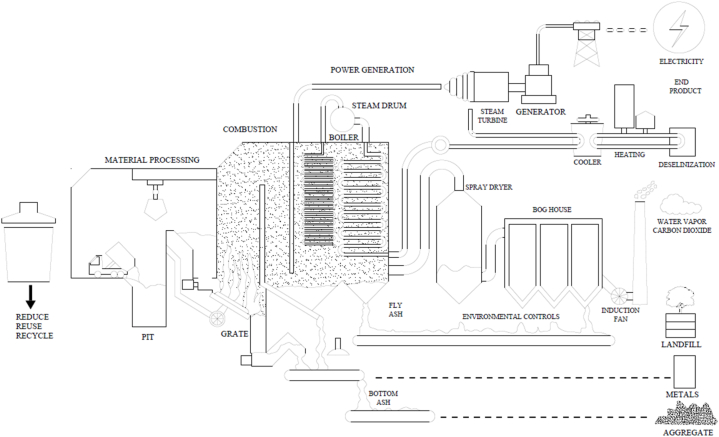
RDF used in coal power plant [62].

One of the most proven ways to decarbonize and deal with waste is through using RDF in thermal power plants as shown in Fig. 10. RDF is an environmentally friendly way of combining RDF with traditional fuels like coal for effective combustion. This is primarily a fractionally separated MSW feed, yielding mainly RDF.

RDF can reduce greenhouse gases as well as cut the cost by 20 % compared to coal in case of power plant emissions [43,44]. Co-firing RDF, in particular, has been said to decrease CO₂ emissions by approximately 30 % with respect to coal-only combustion [48,50]. In power plants the RDF is used as it eliminates loss of waste that goes to landfills and converts this very waste into strong fuel. It is a win-win from the perspective of this planet — it fulfills energy needs while mitigating adverse environmental impacts, as also enhances energy security. RDF suitable for coal power plants aligns with German certification RAL GZ 724, requiring lower chlorine content than cement kilns [51]. In German plants, RDF substitution rates range from 1.8 % to 4 %, maintaining stable operational performance and emissions, consistent with findings by Velis et al. [52].

### 6.3 Utilization of RDF in steel production

Countries like Austria, Germany, and Japan have implemented RDF in steel production as part of broader initiatives to cut carbon emissions and enhance energy efficiency. For instance, in Austria, RDF—with a calorific value of 16–20 MJ/kg—partially substitutes coal in electric arc furnace (EAF) processes, leading to a 20–30 % reduction in CO₂ emissions and significant cost savings due to lower fossil fuel use and reduced landfill needs [53]. However, maintaining high-quality RDF—considering calorific value, moisture, and contaminant levels—is essential, as RDF's variability can disrupt the stable, high temperatures required in steel furnaces. RDF may contain trace elements such as chlorine and heavy metals, potentially forming slag in EAF production if levels are unregulated [48,52]. Furthermore, RDF poses challenges related to combustion and pollutant formation, making a high-performance fuel injection system combined with emission control systems essential for effective compliance with clean combustion regulations. Additionally, EAF fuel injection systems must be adapted to account for the variability in RDF's moisture content and particle size, ensuring reliable feeding and uniform burning [49,50]. In recent years, emission control systems at RDF combustion facilities have been upgraded, with a particular focus on incinerator monitoring and the replacement of existing emission control equipment to meet both EU and Japanese environmental standards [25,28].

Economically, introducing RDF in steel production offers substantial cost reductions. RDF is often cheaper than coal or natural gas and helps minimize waste disposal costs in areas with high landfill taxes, such as Germany and Austria. Steel plants using RDF can save approximately €50–80 per tonne in waste management. Additionally, government incentives like carbon credits or renewable energy certificates can provide further financial benefits for steel producers [30,42,51,53]. However, significant initial investments are required to retrofit steel production infrastructure, ensuring RDF compatibility and regulatory compliance. These investments include advanced fuel processing and emissions control technologies. Public support programs and regulations in many countries also financially encourage RDF adoption in steel production [25,28].

RDF has environmental and economic advantages over other energy sources, such as coal and natural gas. Although coal has a better calorific value (25–30 MJ/kg) and is inherently more consistent in composition, RDF brings down the CO₂ emission by 15–20 % from an existing combustion facility at a typical energy demand meanwhile utilizing non-recyclable waste, which was earlier destined to landfills [30,44,50,53]. The variable matrix of RDF presents operational challenges, such as the need for more complex fuel-feed and combustion systems. While biomass or natural gas has been a more developed application, RDF as fuel for steel production is an area where experiments are still being done to push energy savings and waste management in the circular economy profile.

### 6.4 Utilization of RDF in lime kilns

Lime kilns have been observed to use RDF more frequently, although this is not as well documented as its use in cement and power generation, which seems to eventually become a viable alternative for efficiently utilizing lime production through environmentally friendly fuels/energy. Lime kilns, like cement kilns, operate at elevated temperatures (900–1200 °C) and require fuel with a consistent energy content for efficient limestone calcination. This creates an opportunity for RDF as a substitute for coal or natural gas; however, certain eco-technical challenges arise [49,50,54]. One of the main challenges with RDF in lime kilns is achieving a homogeneous calorific value and moisture content. Most lime kilns require RDF with a calorific value similar to that of coal, around 20–25 MJ/kg, and moisture content in the vicinity of 10–20 % to maintain sustainable operations without disturbing their thermal profile [30,44].

RDF is a complex mix of components, so effective handling of RDF involves special modifications to equipment. It also requires changes in fuel feeding systems for mixed materials and upgrades to combustion chambers operating with higher ash content and contaminants like chlorine (Cl) or sulfur. These process variables are crucial to identify and manage for operational stability [28,32]. Furthermore, ash from RDF can contain heavy metals and residuals that pose challenges for its resourceful management. In addition, lime producers incorporate state-of-the-art ash handling and filtration systems to prevent contamination of the final lime product or harmful emissions.

Lime kilns aim to reduce CO₂ emissions and have demonstrated that using RDF as a substitute for coal could result in 20–30 % lower carbon emissions, depending on its composition and the type of lime kiln [31,41,43]. Nonetheless, due to the variable composition of coke—especially in terms of nitrogen and sulfur compounds—burning RDF has led to higher emissions of NOx (Nitrogen Oxides) and SOx. For example, SOx emissions from RDF can be 5–10 % higher, and CH₄ emissions can be nearly eight times greater compared to natural gas [42,53]. These are not necessarily low-emitting generators, although modern scrubbers and other emission control systems can mitigate these emissions. Not only does using RDF in lime production support sustainability goals by reducing the consumption of finite fossil fuels and diverting materials from landfills, but it also complies with legislative requirements such as the Emission Trading System (ETS), which imposes strict emission limits for carbon reduction on industrial plants like kilns [35,42]. Enforcing these guidelines often requires the installation of continuous emissions monitoring (CEM) systems to maintain RDF combustion within permissible limits. Adhering to these regulations often necessitates the installation of CEM systems to ensure RDF combustion stays within permissible limits [41,50].

The cost of using RDF in lime kilns typically ranges from €20 to €40 per tonne, while coal costs around €50 to a few hundred euros per tonne [30]. Therefore, RDF presents an attractive option for lime producers looking for inexpensive fuel [42]. Additionally, RDF can help reduce waste disposal fees, which can reach up to €100 per tonne in the EU. However, converting lime kilns for RDF combustion requires a significant capital investment, ranging from $500,000 to $2 million, due to the need for specialized fuel injection systems and emission control [40,47]. To offset these costs, governments often provide subsidies and carbon credits as incentives. In the EU, for instance, power plants using RDF can access fines and green energy certificates as additional revenue streams, enhancing the overall economic viability of using RDF instead of landfilling [28,37,53].

Lime kilns are essential for converting limestone into calcium oxide, commonly known as quicklime, which is a critical chemical compound used in various industries [55]. In Europe, quicklime is primarily utilized in metal processing, but it also plays important roles in environmental protection, agriculture, forestry, and as a binder in construction. Additionally, it is widely used in the chemicals, paper, food, and glass industries. In 2021, global lime production reached 430 million tons [56]. Traditionally, quicklime production relies on fuels such as coal, coke, and natural gas.

While there is limited documentation on RDF use in lime kilns compared to cement kilns, it remains a promising alternative fuel [50,53]. The European Commission reports that solid waste is already partially utilized in the lime industry, with a German plant achieving a 10 % thermal substitution rate in 2006 [48,51]. The same report indicates that up to 60 % substitution is technically feasible, though quality assurance is essential for increased waste use. Key challenges include the physical and chemical properties of waste and fuel availability. In Japan, Nomura [51] found that RDF could produce coke for lime kilns, akin to its use in steel production, while gasified RDF can replace coke [57]. The German quality certification RAL GZ 724 evaluates RDF properties suitable for lime kilns [51,52]. Quality requirements for RDF in lime kilns are detailed in Table 9.

## 7 Environmental impact of using RDF as a fuel

RDFs must be assessed around essential measurements, such as pollution and GHG emissions, resource efficiency, and lack of unrenewable sources. Together, these effects demonstrate how RDF could be a more environmentally friendly fuel by substituting fossil fuels like coal and making part of the circular economy.

### 7.1 Air quality and emissions

Burning RDF, being a proper waste-to-energy technology, has the potential to deliver 50 % less GHG emissions than more common fossil fuels that resemble coal and natural gas in specific terms. According to the Kuila et al. [58], when RDF replaces coal in energy generation, a reduction of CO₂ emissions is achieved between 20 and 30 %, depending on the composition and quality of this one. This is mainly because RDF employs a waste that has already absorbed carbon during its life cycle and thus involves less net CO₂ emissions when burned. Nonetheless, the combustion of RDF can produce pollutants (e.g., NOx and SOx), as well as particulate matter PM, especially when the RDF is high in chlorine or other contaminants. These are best controlled with the addition of advanced exhaust gas cleaning systems: scrubbers, bag filters and electrostatic precipitators, which have to be used in combination with one another for this purpose. Table 10 compares the significant emissions from RDF to coal and natural gas. Table 10 for RDF shows the emissions CO₂ and SOx to be lower than coal, while depending on waste composition, it may still require some form of emission management (specifically NOx and particulate matter).

**Table 10:** Emissions comparison of RDF, coal, and natural gas [28,30,42].

Fuel	CO₂ Emissions (kg/GJ)	NO_x_ Emissions (g/MJ)	SO_x_ Emissions (g/MJ)	Particulate Matter (g/MJ)
RDF	50–70	0.10–0.25	0.05–0.15	0.03–0.10
Coal	80–100	0.30–0.50	0.50–1.50	0.10–0.50
Natural Gas	55–65	0.05–0.10	0.01–0.02	0.01–0.03

### 7.2 Resource conservation and circular economy

**Fig. 11:**
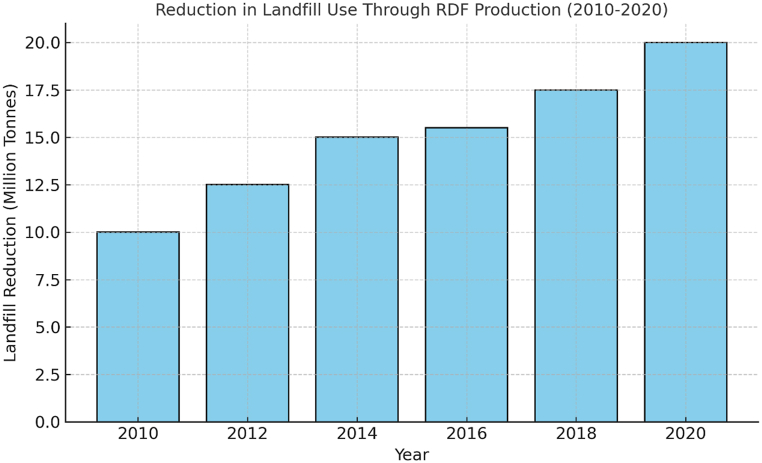
Reduction in landfill use through RDF production [59].

RDF plays a vital role in conserving valuable resources by replacing non-renewable fossil fuels with renewable energy generated from waste. This transition fosters a circular economy by repurposing refuse that would otherwise be discarded. Waste-to-Energy technologies can significantly reduce landfill use and the associated emissions of methane, a potent greenhouse gas, by diverting up to 60 % of municipal solid waste (MSW) from landfills [28,43]. As landfill space becomes increasingly scarce, RDF offers environmental benefits through waste diversion. For instance, RDF production has potentially prevented 15–20 million tonnes of landfill expansion annually in Europe, resulting in significant savings from biodegradables decomposed through anaerobic processes, thereby reducing methane emissions from organic waste. This shift away from landfilling aligns with broader sustainability goals, as methane is 28–36 times more effective than CO₂ at trapping heat over a 100-year period. Fig. 11 illustrates the amount of RDF diverted from landfills over time, highlighting its impact on waste diversion and landfill reduction, as well as the breakdown of RDF utilization.

### 7.3 Comparative analysis: RDF vs. other alternative fuels

Compared to other alternative fuels such as biomass, biogas, and liquid biofuels (bioethanol), RDF has some significant advantages for resource conservation with respect to waste prevention. Although renewable, this does compete with food crops and can cause deforestation. RDF, conversely, is derived from waste material that would otherwise be disposed of, meaning it is more resource-efficient in itself [28,32,36]. While biogas and liquid biofuels are typically renewable, the production processes associated with these fuels can be far more complex than RDF manufacturing which may lead to higher costs and energy inputs. Furthermore, RDF can better serve industrial uses like cement kilns or power generation with its more consistent energy output than biogas, which might vary according to feedstock composition. Table 11 represents the environmental impact compared amongst various alternative fuels and RDF based on emissions and rebound effects with different alternatives for RDF [53].

**Table 11:** Environmental comparison of RDF and alternative fuels [28,30,42,53,54].

Fuel	CO₂ Emissions (kg/GJ)	Waste Reduction (%)	Land Use Impact	Scalability
RDF	50–70	60 %	Minimal (waste-based)	High
Biomass	30–50	N/A	High (land required)	Medium
Biogas	20–30	20–30 %	Low	Low (localized)
Liquid Biofuels	40–60	N/A	Medium (crop-based)	Medium

RDF's scalability makes it a practical choice for regions with significant waste generation. In contrast, its environmental benefits—particularly in terms of waste diversion and reduced reliance on virgin resources—align with global sustainability targets. RDF provides many environmental advantages compared to fossil fuels, particularly in significantly reducing greenhouse gas emissions. Co-firing RDF with coal in a power plant can reduce CO2 emissions by 30–50 % compared to using only coal [50,53]. Furthermore, using RDF facilitates diverting large quantities of MSW from landfills, thereby reducing methane emissions—a potent greenhouse gas produced by degrading organic matter [12,34,38]. Recycling and upcycling are also beneficial methods because RDF transforms refuse into a high-quality resource, relieving environmental stress on waste management. Japan and Germany were pioneers in RDF adoption. The thermal substitution rate in cement plants using RDF varies from 1.8 % to 4 %, with a maximum of up to 60 %, without a notable effect on operational performance or emissions [52,53]. This significantly reduces landfilling, particularly for energy-rich waste fractions.

Conversely, traditional fossil fuels like coal and natural gas have high carbon footprints and significantly contribute to air pollution. Coal combustion emits CO_2_ (the number one greenhouse gas), sulfur oxides, nitrogen oxides, and particulate matter, all degrading air quality and polluting the environment, often leading to significant human harm. Unlike raw waste, RDF from this process would likely contain lower sulfur and nitrogen content than its fresh feedstock [36,50,53], which implies NOx and SOx reductions. Moreover, RDF is appropriate as it is more efficient and can afford to regain energy economically. When combined with a gas micro-turbine, RDF has the potential to produce very high quantities of electricity, albeit with an extensive reduction in emissions compared to standard fossil-fuel-based power generation [12,34,45]. This solution resonates with the broader challenges we face on a global scale in sustainability, as it supports new sustainable waste-to-energy technologies, which are essential to moving populations away from fossil energy and feedstock use, also impacting climate change.

Table 12 highlights RDF's environmental and economic aspects vs. conventional fossil fuel use. The table summarises data on solid fuels' CO_2_ emission rate, calorific value, and sulfur/nitrogen content. It also shows additional costs such as pollutant discharge loads, energy resource savings degrees, and reliability. This study shows that RDF has superior benefits to conventional fossil fuels like Coal and Pet coke in reducing GHG emissions and supporting sustainable waste management solutions.

**Table 12:** Comparison of RDF and traditional fuels.

Aspect	RDF	Traditional Fuels (Coal, Petcoke)
CO_2_ Emissions (kg CO_2_/GJ)	8.7 [50]	Coal: 96, Petcoke: 101 [50]
Calorific Value (MJ/kg)	18-24 [9]	Coal: 20–25, Petcoke: 33–35 [9]
Sulfur Content	Low [60]	High [61]
Nitrogen Content	Low [60]	High [61]
Pollutant Emissions	Lower NO_x_ and SO_x_ emissions [60]	Higher NO_x_ and SO_x_ emissions [61]
Cost	Lower cost due to waste management benefits [9]	Higher cost due to extraction and processing [9]
Resource Efficiency	Utilizes waste materials, contributing to circular economy [50]	Extracts finite natural resources [50]
Energy Security	Enhances energy security by diversifying fuel sources [9]	Relies on finite fossil fuel reserves [9]

## 8 Conclusions

This study offers significant insights into the use of RDF as a greener alternative to traditional fossil fuels, particularly within waste management, renewable energy, and industrial sectors. We focused on five strategic locations in Uttar Pradesh to explore RDF application in cement production, power generation, and waste-to-energy plants. Our results demonstrate that RDF can substantially reduce global warming potential, decrease reliance on non-renewable resources, and divert waste from landfills, thereby contributing to international sustainability targets and the circular economy. Unlike previous studies that primarily examined developed nations, this research highlights the potential for implementing RDF practices in developing countries like India, where waste generation is rapidly increasing. The findings underscore RDF's viability at scale, with acceptable calorific values and moderate emission levels. Methodologically, the study introduces a broad comparative analysis of RDF produced from diverse feedstock mixes and regions with varying compositions, enhancing our understanding of how local factors influence constituent variability. This multi-site approach distinguishes our work and provides valuable lessons applicable to other regions facing similar waste management and energy supply challenges.

The results underscore important policy and industry considerations. This is where the need for RDF arises, as countries aim to reduce their carbon footprint and landfill impacts. Policymakers are urged to financially support RDF production through tax exemptions, feed-in tariffs, and carbon credits to accelerate business acceptance. It is crucial to establish quality criteria for RDF, particularly regarding calorific value and moisture content regulation. This research contributes by highlighting the use of RDF in energy systems as a means to improve environmental resilience and reduce fuel costs (along with waste disposal fees), all of which contribute to financial stability. At a time when cities worldwide are grappling with increasing waste and confronting climate change, this research highlights an urgent local opportunity. As landfills continue to fill up, RDF—a fuel produced by processing waste materials—can serve as a viable waste-to-energy solution for managing discarded items. Additionally, RDF is greener than merely burying trash, especially as we move closer to decarbonization and a transition toward clean energy, and its low CO₂ emissions, which also help reduce reliance on fossil fuels. This indicates that RDF is crucial for addressing waste management challenges and enhancing the clean fuel mix, positioning it as a key player in sustainable energy and addressing our environmental needs.

## 9 Future recommendation

It is recommended that future research on RDF incorporate methodological refinements to enhance precision and utility. To achieve this, employing more sophisticated analytical techniques, such as spectroscopic methods, would provide higher-quality data for RDF characterization. Future studies should survey larger samples with greater variety from different regions to investigate socio-economic differences and seasonal impacts on waste composition.

Since the quality of RDF must remain consistent, stricter waste segregation practices should be implemented with the support of automation. Research should explore the global suitability of RDF, considering both industrialized countries with high emissions standards and developing economies where waste infrastructure is most needed. It is crucial for regions considering RDF technologies to understand their scalability to meet local energy demands.

The resource efficiency and sustainability principles of circular economy models should support the inclusion of RDF in waste-to-energy systems. Further work is needed to develop policy frameworks compatible with decentralized RDF deployment, such as carbon pricing or renewable energy credits that can enhance the market competitiveness of alternatives. Engineers must collaborate with environmental scientists, economists, and social scientists to create interdisciplinary approaches for policymaking and new technologies.

LCA should be utilized to determine the environmental impacts of RDF, and full life-cycle cost analyses addressing factors like greenhouse gas mitigation benefits or avoided landfill burdens are also necessary. Establishing key performance indicators (KPIs) and continuous monitoring will help ensure RDF technologies evolve in response to regulatory and market changes. In doing so, pre-treatment processes will facilitate innovation and improvement, reinforcing RDF as a suitable choice during this transitional period for sustainable waste management.

## CRediT authorship contribution statement

**Utsav Sharma:** Writing – original draft, Validation, Software, Methodology, Investigation, Formal analysis, Conceptualization. **Dayanand Sharma:** Writing – review & editing, Visualization, Software, Data curation, Conceptualization. **Amit Kumar:** Writing – review & editing, Visualization, Software, Methodology, Conceptualization. **Tushar Bansal:** Writing – review & editing, Visualization, Validation, Software, Conceptualization. **Ankit Agarwal:** Writing – review & editing, Visualization, Software, Investigation, Conceptualization. **Shudhanshu Kumar:** Writing – review & editing, Software, Methodology, Investigation, Data curation. **Abid Hussian:** Writing – review & editing, Visualization, Validation, Software, Methodology. **Hesam Kamyab:** Writing – review & editing, Visualization, Validation, Supervision, Formal analysis. **Moinul Haq:** Writing – review & editing, Visualization, Validation, Software, Conceptualization.

## Declaration of competing interest

The authors declare that they have no known competing financial interests or personal relationships that could have appeared to influence the work reported in this paper.
